# Controlling the Dynamic Behavior of Microposts in Solution via Diffusion–Convection

**DOI:** 10.1021/acs.langmuir.4c04567

**Published:** 2025-03-05

**Authors:** Moslem Moradi, Oleg E. Shklyaev, Anna C. Balazs

**Affiliations:** Department of Chemical Engineering, University of Pittsburgh, Pittsburgh, Pennsylvania 15261, United States

## Abstract

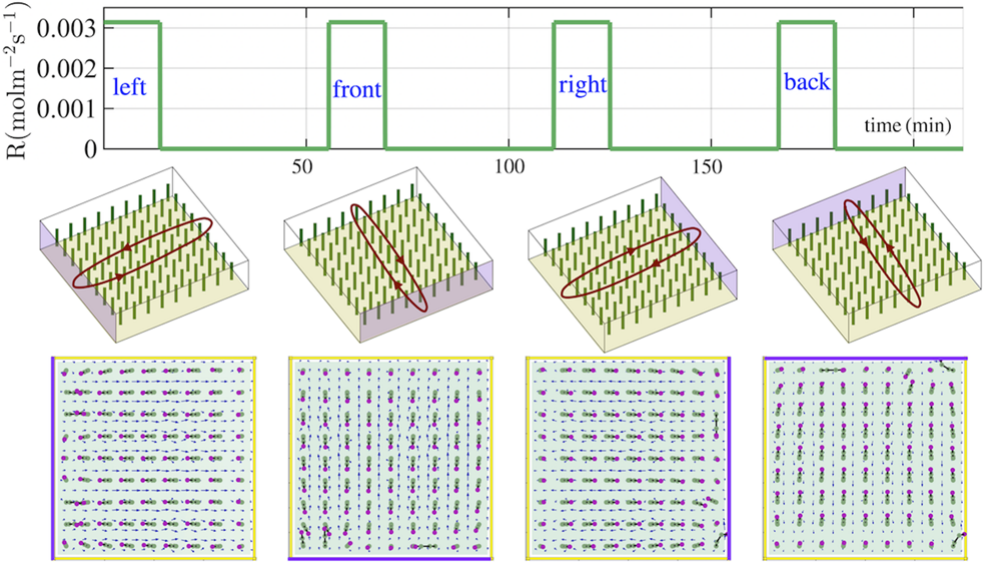

Solutal buoyancy forces in solution arise from density gradients, which occur when the reactants and products of a chemical reaction occupy different volumes in the fluid. These forces drive fluids to spontaneously perform self-directed mechanical work such as shaping and organizing flexible objects in fluid-filled microchambers. Here, we use theory and simulation to show that chemical reactions are not necessary to generate useful solutal buoyancy forces; it is sufficient to just add reactants to aqueous solutions that have a different mass-to-volume ratio than water to drive such spontaneous mechanical action. To demonstrate this behavior, we model arrays of tethered, nonreactive posts in a fluid-filled chamber. Relatively dense chemicals released from the chamber’s side walls diffuse into the solution and generate buoyancy-driven flows, which spontaneously trigger the posts to undergo collective dynamics. The posts’ dynamics can be controllably programmed by staging the sequence of chemical release from the different walls. With chemically active posts within the array, turning on and off the influx of chemicals from the side walls leads to propagating waves that drive the posts to exhibit biomimetic coordinated motion. The introduction of cascade reactions dynamically shifts the direction of wave propagation. Our findings show how diffusion–convection and diffusion–reaction–convection processes can be used to regulate nonequilibrium spatiotemporal behavior in fluidic systems. This level of control is vital for creating portable microfluidic devices that operate without external power sources and thus function in remote or resource-poor locations.

## I Introduction

Complex patterns and self-organized structures can emerge in fluid-filled microchambers when a chemical reaction in the solution generates products that have a different volume than reactants.^1−5^ These differences in volume lead to local density gradients and resultant forces (referred to as “solutal buoyancy” forces), which act on the fluid and thereby trigger the fluid’s spontaneous motion and rich spatiotemporal behavior. The flowing fluid in turn can autonomously perform mechanical work as it transports particulates or reconfigures flexible objects in the solution.^1−4^ Using theory and simulation, we previously focused on fluid-filled chambers containing flexible, chemically reactive posts and showed that chemical reactions in the solution produced solutal buoyancy forces, which regulated the reconfiguration and biomimetic communication in the array of immersed posts.^4^ We now focus on fluidic chambers containing passive posts, which do not participate in chemical reactions, to pinpoint other mechanisms for controlling the dynamic interactions among such compliant structures. In the cases considered here, chemicals diffuse into the surrounding solution from the side walls of the chamber. These chemicals can inherently have a higher (or lower) density than water. We show that the resulting density differences in the passive systems also generate solutal buoyancy forces and convection of the fluid. We further demonstrate that this combination of diffusion and convection is sufficient to drive biomimetic communication among passive flexible posts. Furthermore, varying the spatial arrangement of the passive posts and the temporal staging of the chemical release from the walls provide an effective means of regulating the shape and structure formation in the posts. While the effects of diffusion–convection on the dynamic behavior of immersed, hard particles have been studied,^6−29^ to the best of our knowledge, the combined influence of diffusion and convection on immersed deformable objects remains relatively unknown. Consequently, researchers may be overlooking opportunities to simultaneously control the shape and life-like messaging in synthetic materials systems.

Solutal buoyancy is a bulk phenomenon;^30−33^ consequently, fluid diffusing from the side walls of the chamber impacts the entirety of the flexible posts, which can bend and sway through fluid–structure interactions. (In contrast, diffusioosmosis can propel fluids due to chemical concentration gradients at immobile walls or mobile particles, leading to forces that are primarily operative near a surface.) The posts, in turn, exert an opposing force on the fluid, introducing additional fluid–structure interactions. As shown below, the cross-talk between hydrodynamics and fluid–structure interactions can be tailored to provide significant control over the dynamic behavior of the immersed posts, especially when the diffusion of chemicals occurs from multiple side walls in this system. In the latter case, the movement and orientation of the posts can be programmed and reprogrammed by varying the sequence in which chemicals are released from the walls.

Two recent studies indicate the feasibility of our studies and highlight the rich dynamics that emerge when chemical diffusion contributes to solutal buoyancy effects. Specifically, Das et al. introduced a gel reservoir at one end of a chamber where the entire bottom surface was coated by a single catalyst.^34^ The diffusion of chemicals from the gel generated a nonuniform density gradient along the length of the chamber that propelled the flow. The subsequent reaction between the diffusing chemicals and the catalyst-coated surface generated further density gradients, which amplified the effects of the initial diffusive flow. The sum of these solutal forces enabled the convective flow in the chamber to controllably transport and deliver microparticles at specific locations in the microchambers. Additionally, Maiti et al. created controllable fluidic “circuits”, using enzymes encased in immobile porous gels, which were placed at the bottom of a fluid-filled chamber.^35^ Since enzymatic reactions are highly selective, fluid flow occurs only when the right reactant in the solution has diffused to the appropriate gel container and could thereby trigger the solutal buoyancy mechanism. The researchers further underscored the role of chemical diffusion in the enzyme-containing gels by showing that the circuit layout and flow direction of each constituent stream could be controlled through the number and placement of enzyme-containing gels in the chamber.

The latter experiments point to the achievability of the scenarios described below. In the first set of simulations in this study, density gradients are created when reactants denser than water diffuse into the solution through the side wall(s) of a microchamber. In the second set of simulations, we coated some posts in the tethered array with specific enzymes to introduce buoyancy effects due to chemical reactions. These studies allowed us to devise cases where the sequential occurrence of diffusion, reaction, and convection drives the system to undergo multistaged reactions, leading to control over the spatial and temporal behavior in the fluidic chamber.

Our findings on the dynamic behavior of the immersed tethered posts can facilitate the development of microfluidic platforms that do not require the construction of new internal walls for each new application and thereby reduce the cost of fabricating multipurpose devices. The ease of using these platforms can also facilitate the creation of portable, autonomous fluidic devices for resource-poor or remote locations. Namely, in all cases considered here, the systems did not require external pumps to propel the fluid motion but were driven simply by the introduction of chemicals into the solution. On a fundamental level, the studies can reveal the role of hydrodynamics and fluid–structure interactions in the propagation and interpretation of chemical signals through fluidic networks in living systems. Biological signal processing may involve complex biochemical “machinery”, but diffusion, reaction, and convection arise inherently in fluid-filled channels and hence may play a crucial role in the biological processes.

## II Materials and Methods

In a fluid-filled chamber, the chemical influx (glucose) through the side walls of the microchamber generates local density changes (increases), which generate convective flows, primarily due to solutal buoyancy. Therefore, the fluid motion is coupled to the chemical composition of the solution within the microchamber. In our model, local variations in density ρ(**r**, *t*) of the aqueous solution depend on the concentration *C*_*i*_(**r**, *t*) of *N* reactants at time *t* as , where ρ_0_ is the solvent density and  are the corresponding solutal expansion coefficients, which characterize local density variations in the solution. We ignore the effect of thermal buoyancy since thermal expansion coefficients are significantly smaller than the solutal expansion coefficients for the chemical reactions considered here.^36^ The fluid flow is generated by the buoyancy force  (with vector **g** denoting the gravitational acceleration). This buoyancy force drives the spontaneous motion of the fluid and consequently moves immersed particles and deforms immersed elastic posts. The immersed particles are modeled as spheres of radius *b*, with density ρ_*m*_ and experiencing gravitational force .

The simulation domain, Ω = {(*x*, *y*, *z*): 0 ≤ *x* ≤ *L*_*x*_,0 ≤ *y* ≤ *L*_*y*_,0 ≤ *z* ≤ *H*}, is a rectangular box with horizontal dimensions *L*_*x*_ and *L*_*y*_ and height *H*. The elastic post is modeled as a linear chain of *M* beads, described by positions, **r***_k_*, that are interconnected by elastic rods. The nodes of active posts are uniformly coated with a catalyst that generates the buoyancy force. The passive posts are uncoated, so the buoyancy forces originate solely from the reactions promoted by the surface-coated active posts. The movements of the spherical beads (nodes) forming the posts are described as
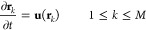
1where **u**(**r**) is the local fluid velocity at each bead. Each bead in a chain experiences forces due to steric repulsion from the other beads, **F**_s_^nn^, and from the side walls of the channel, **F**_s_^nw^. The steric repulsion force, **F**_s_(**r**) = −∇ *U*(*r*), is computed from the Morse potential (SI Appendix). The bonds between the beads experience an elastic force, **F**_el_^n^, which is characterized by the stretching (κ_*s*_) and bending (κ_*b*_) moduli and is governed by the linear constitutive relations for a Kirchhoff rod.^37^ The first bead in each chain is located at a height *d* from the bottom wall and is anchored to that wall by a spring force at *z* = *d*. We assume that the density of the posts is the same as that of the solvent (ρ_0_), so that the posts are neutrally buoyant. To conserve momentum exchange between the post and the fluid, the forces acting on each bead are balanced by the hydrodynamic drag force **F**^**h**^ = −(**F**_el_^n^ + ∑**F**_s_^nn^ + ∑**F**_s_^nw^).

The fluid dynamics in the chamber are described by the respective continuity, Navier–Stokes equations (in Bossinesq approximation^38^), and reaction–diffusion equations
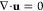
2
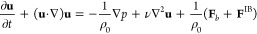
3
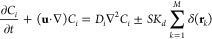
4Here, **u** is the fluid velocity, *p* is the fluid pressure, and ν is the kinematic viscosity. The body forces acting on the fluid have contributions from the solutal buoyancy force, **F***_b_*, and the force due to deformations of elastic posts that act on the fluid, **F**^IB^ = −∑**F**^h^, which is calculated via the immersed boundary method^37^ (IBM), providing fluid–structure interactions between the solution and the elastic posts.

The chemicals are consumed or produced at the position of the enzyme-coated bead **r***_k_* of the post with a reaction rate given by  where *S* is the area coated by the catalyst. (The “ ± ” sign in eq 4 represents either production or consumption of the reactants and products.) We assume that the catalytic reaction on the post follows the Michaelis–Menten kinetics,^39^ where the rate of consumption per unit area of the catalytic coverage is given by
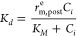
5Here, *r*_m,post_^e^ = *k*_e_[*E*] (in units of mol m^-2^ s^-1^) incorporates the maximum reaction rate per molecule of enzyme, *k*_e_, with areal enzyme concentration [*E*], and *K*_*M*_ (in units of molarity, M) is the Michaelis constant. Both reactants and products undergo diffusion within the solution and are advected by the generated fluid flow.

We prescribe no-slip boundary conditions for the fluid velocity, **u**(∂Ω) = **0**, at the confining solid walls. For chemicals *C*_*i*_, we impose no-flux boundary conditions,  and constant chemical influx, , at specified (side)walls of the microchamber, where ∂Ω_1_ and ∂Ω_2_ are the side walls with no-flux and constant influx, respectively, (∂Ω_1_ ∪ ∂Ω_2_ = ∂Ω). The set of governing equations (eqs 1–4), along with these boundary conditions for the fluid velocity and chemical concentrations, is solved numerically.

The continuity and Navier–Stokes equations are solved using the lattice Boltzmann method^40^ (LBM) with a single relaxation time D3Q19 scheme.^41^ A finite difference approach with a forward-time central-space (FTCS) scheme is used to solve the advection–reaction diffusion of chemicals. The size of the computational domain is 40Δ*x* × 40Δ*x* × 10Δ*x* where the lattice Boltzmann unit Δ*x* is 100 μm. The time step of the simulation is Δ*t* = 1.67 × 10^–3^ s. In the IB approach, each of the discretized nodes of the post is treated as a sphere with radius *a* ≈ 1.3Δ*x*. Each post is discretized into *M* = 5 beads with the equilibrium distance between the beads set to 1.75Δ*x*. Moreover, we place the first bead at a distance Δ*x* from the wall, so that the total length of the post is *L* = 8Δ*x*.

In the simulations, we specify a value for the bending stiffness of the post (κ_*b*_). The values for bending energy are extracted from the values of corresponding flexural rigidity (B) of soft flexible films provided in the literature.^42−44^ The latter materials could be used for the experimental realization of our model system. To deduce necessary parameters, we take experimental values of B and use the one-dimensional linear beam equation^45^ to analytically describe a beam clamped at one end (as for the tethered posts) and subjected to a given transversal force *F* at the free end. Then we performed simulations for the same force *F* and adjusted values of κ_*b*_ to obtain the same transversal displacement as obtained in the theoretical approach. The procedure provides the correlation between the simulation parameters κ_*b*_ and experimentally used flexural rigidities B as described in the SI Appendix.

The parameters relevant to the chemical reactions on the surface of the catalyst-coated posts are given in the SI Appendix, Tables S1 and S2. We estimate that the chemical influx in our simulations is approximately . This value is comparable to values obtained in experiments for gradient-driven fluid flow in microfluidic devices.^12^ In our simulations, we use reaction rates *r*_m, post_^e^ that are comparable to the experimental values reported by Sengupta et al.^46^

## III Results and Discussion

We start the discussion with fluid-filled chambers, where solutal buoyancy effects occur without a chemical reaction. Here, the diffusion of chemicals from a reservoir (located inside the side walls of the chamber) introduces an influx of chemicals. If a dissolved, diffusing chemical occupies a molecular volume different from that of water, the presence of this chemical creates a density gradient and subsequent solutal buoyance effects in the solution. The buoyancy-driven flow and flexible posts in the solution undergo fluid–structure interactions, which lead to feedback in the system and the remarkable behavior described below.

### III.A Effects of Solutal Buoyancy without Chemical Reactions: Diffusion–Convection

#### III.A.1 Chamber without Posts

**Figure 1:**
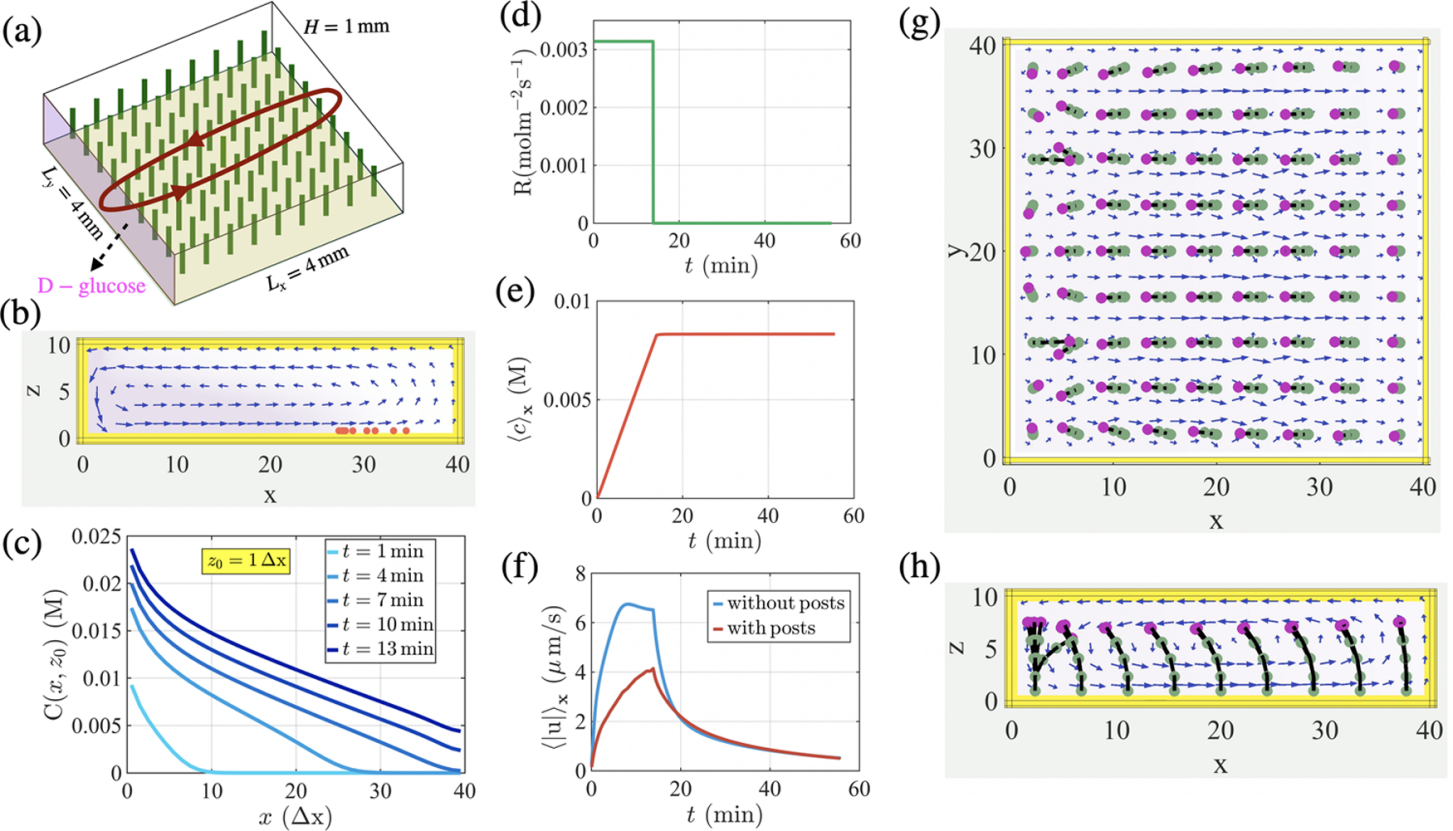
Convective flow generated by a chemical influx from left wall of a microchamber. (a) Schematic view of a fluid-filled chamber (of dimensions 40Δ*x* × 40Δ*x* × 10Δ*x* where Δ*x* = 0.1 mm is the lattice Boltzmann unit) containing a 9 × 9 array of flexible posts, where the reagent (released by a more dense d-glucose gel) enters the domain through the left side wall at *x* = 0. (b) Side view of the flow generated by the influx of chemicals through the left side wall where the heavier tracer particles move to the right of the microchamber. (c) Concentration field as a function of lateral distance from the left side wall, *x*, at different times at height of *z* = 0.1 mm. (d) Chemical influx, which is set to *R* = −*D*∂*C*/∂*x*(*x*=0) = 3.14×10^–3^ mol m^–2^ s^–1^ is turned on for 14 min and then turned off. (e) Average glucose concentration in the domain as a function of time. (f) Average velocity field in the domain as a function of time with and without the presence of posts. Top view (g) and side view (h) of simulation result at time *t* = 10 min, showing the direction of the posts pointing toward the left wall.

We perform simulations in a rectangular chamber that is 4 mm in each lateral direction, *L*, and has a height of *H* = 1 mm (Figure 1a). The domain contains a 9 × 9 array of vertically oriented flexible, passive posts (shown in green in Figure 1a) tethered by one end to the bottom surface. The length and diameter of the posts are *L* = 0.8 mm and *w* = 0.26 mm, respectively.

To understand how chemicals that enter the domain affect the configuration of the posts, we first consider the situation without the surface-anchored flexible posts within the chamber. (The chamber without the posts is not shown here, as it can be easily visualized as the chamber without the green pillars in Figure 1a.) The flow in the domain is generated through the following mechanism. Reagents diffuse into the solution from the left side wall (shaded pink in Figure 1a) at a constant rate, while the other walls are assumed to be impermeable to the reagent. The reagent in the solution is taken to be d-glucose, which is denser than water. Therefore, the glucose-rich solution near the left wall sinks to the bottom of the chamber, and the fluid flows away from the source (left to right; Figure 1b). Due to the continuity of the fluid in the closed domain, the lighter, glucose-poor solution at the opposite, right wall rises upward and is constrained to move along the top confining surface back to the left wall. We refer to this cycle of fluid motion as outward flow.

The distribution of the dissolved glucose is illustrated in Figure 1c, which shows the glucose concentration as a function of lateral distance (*x*) at different times for a chosen domain height of *z*_0_ = 0.1 mm. The glucose concentration at the left wall is higher for longer times, as indicated by the gradual variation in color of the lines from light blue to dark blue. Since the influx from the left wall occurs at a constant rate, waiting longer times yields a higher initial glucose concentration at that wall. Diffusion and the buoyancy-driven fluid flow (generated by the differences in molecular volume between the solute and water) transport glucose to the right along the bottom.

Figure 1d–1f characterizes the stages in this dynamic process. First, the chemical influx from the left wall is held fixed for some time, Δ*t*_on_, as shown in Figure 1d. As the chemical enters the domain, its average concentration increases linearly with time (Figure 1e), with the average velocity reaching a value of |**u**| ∼ 6.5 μm/s after 8 min (Figure 1f). The mechanical work performed by the generated flow can be visualized through the motion of submerged microparticles (shown in red in Figure 1b), which first sediment to the bottom due to gravity and are then dragged by the flow along the bottom toward the right wall.

#### III.A.2 Chamber with Posts

When an array of passive, elastic posts (shown in green) is attached to the bottom wall, each post experiences fluid drag imposed by the moving fluid. As a result, after a transition period (during which the posts reorient), these posts adopt the configurations indicated by the top and side projections shown in Figure 1g,1h, respectively. To aid the reader in visualizing the posts’ orientations, the top bead of each post is colored magenta, while the body is shown in green. The top view (Figure 1g) reveals that the posts are tilted by the flow toward the left side wall. This tilt results from the fluid drag imposed by the counterclockwise circulating flow (shown in Figure 1h) on the top free beads (and hence the rest of the tethered chain).

The above sequence of events is analogous to events in biological chemo-mechanical transduction, where chemical reactions release energy that is utilized to perform mechanical work. In the synthetic system considered here, chemo-mechanical transduction is triggered by the influx of dense chemicals. The resulting density gradients give rise to solutal buoyancy forces, which act along the length of the post and perform the mechanical work of reorienting the posts. As explained above, the posts reorient toward the chemical source and hence operate as “pointers” to identify the side wall releasing the reagents.

**Figure 2:**
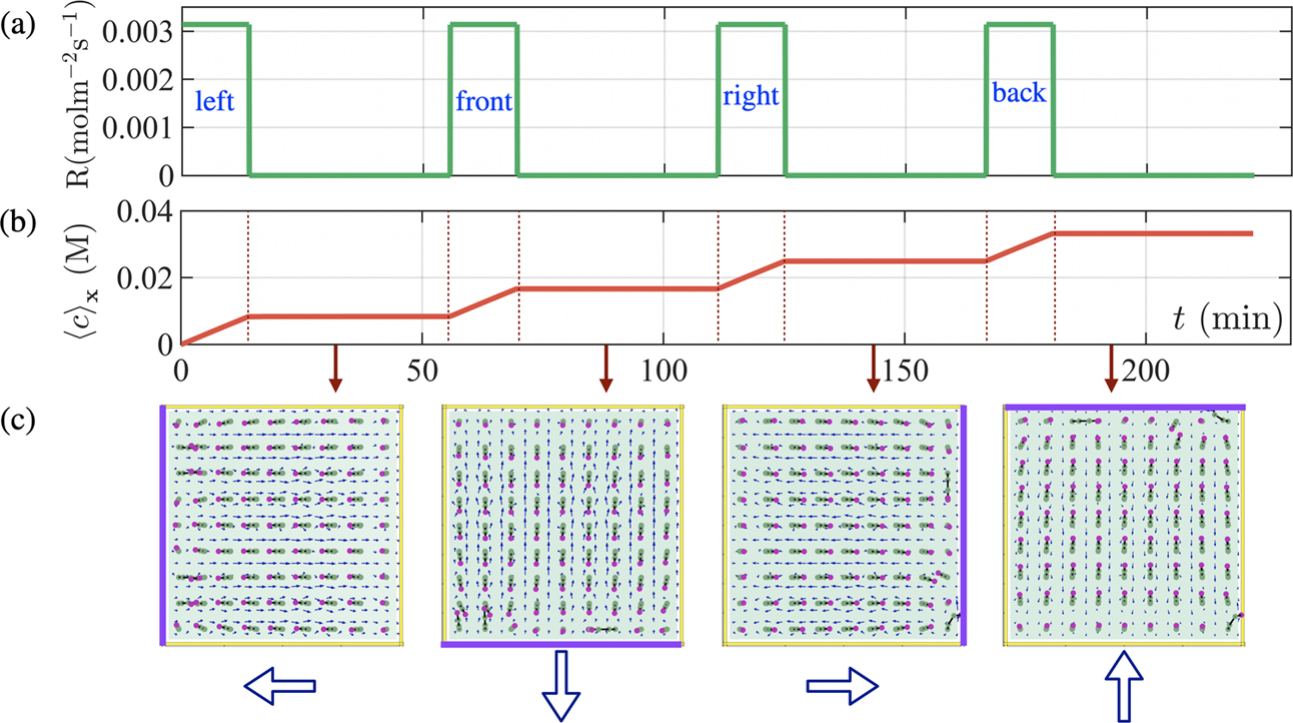
Convective flow generated by introducing consecutive constant chemical influx from all the side walls of a microchamber. (a) Chemical influx, which is set to *R* = 3.14 × 10^–3^ mol m^–2^ s^–1^ is turned on for Δ*t*_on_ = 14 min and then turned off for Δ*t*_off_ = 42 min consecutively, for each side wall at *x* = 0, *y* = 0, *x* = 4 mm and *y* = 4 mm, respectively. (b) Average glucose in the domain as a function of time. (c) Simulation result, showing the direction of the posts pointing to the left, front, right and back side walls at each on–off period of the glucose influx.

The above mechanistic steps can be used to dynamically tailor the orientation of the entire post array. To demonstrate the latter level of control, we impose chemical influx sequentially from all four side walls. Figure 2a shows time periods during which the chemical influx is turned “on” (Δ*t*_on_) and “off” (Δ*t*_off_) at the first, second, third, and fourth walls, respectively. The corresponding increase in the average chemical concentration as a function of time is plotted in Figure 2b. The chemical influx at the corresponding walls generates solutal buoyancy forces and corresponding fluid flows, which drag the immersed, elastic posts and force them to bend. Figure 2c shows the top views of the simulation domain with the reconfigured posts, which reflect events occurring at one of the side walls. We refer to a wall that releases reagents as chemically active; the latter walls are highlighted by purple lines. The arrow below each panel in Figure 2c indicates the final post orientation in the array.

The sequence in which chemicals are released from the active walls and the duration of the active “on” stage can be tailored to impart arrays with different functionalities. The lateral sizes of the domain (∼4 mm) and the post lengths (∼1 mm) are of sufficient scale to be seen by the naked eye. Looking down from the top, the domains reveal that they can act as millimeter-sized display “screens” that reveal the presence of dense chemicals and thereby perform as chemical sensors.

**Figure 3:**
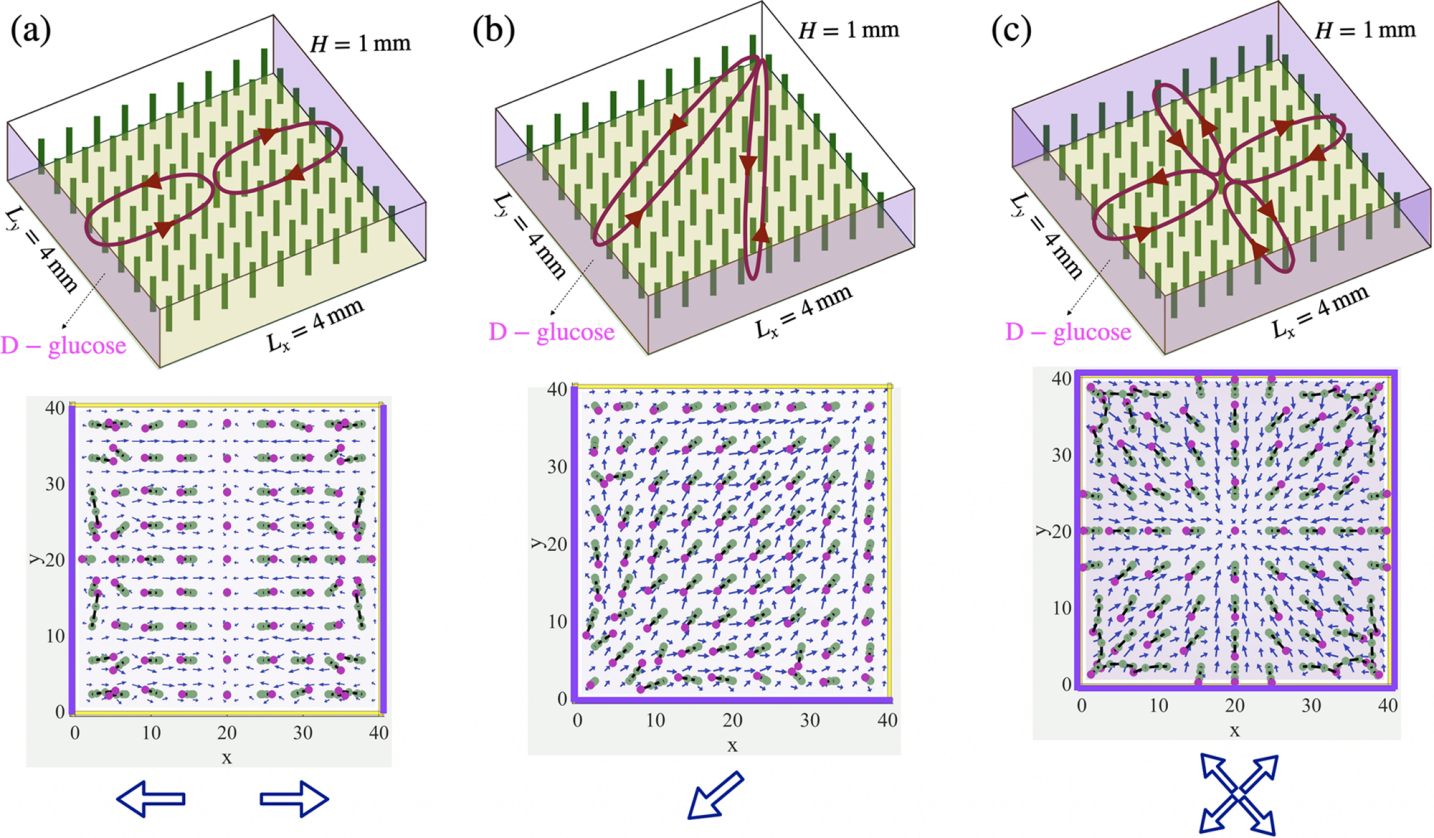
Convective flow generated by constant chemical influx from two and four side walls of a microchamber. Top: schematic view of a fluid-filled chamber containing a 9 × 9 array of flexible posts, where reagent (released by a more dense d-glucose gel) enters the domain through (a) two opposing side walls at *x* = 0 and *x* = 4 mm, (b) two nearby side walls at *x* = 0 and *y* = 0, and (c) all the side walls; bottom: simulation result, showing the direction of the posts pointing toward the left and right side walls (a), lower nearby side walls (b), and all of the side walls (c). The chemical influx rates are set to *R*/2 for (a) and (b), and *R*/4 for (c), where *R* is *R* = 3.14 × 10^–3^ mol m^–2^ s^–1^.

More complex post patterns can be achieved by allowing multiple walls to release reagents simultaneously. Figure 3a shows the pattern that results from the simultaneous release of reagents from two opposing side walls (shaded in pink on the top panel of Figure 3a). In this case, the chemicals diffusing from the left and right walls generate two counter-rotating fluid vortices, which move the fluid toward the respective walls along the top of the domain. This circulating flow drags the post to tilt toward the respective active walls, thereby forming two domains in which the posts are oriented either to the left or to the right, as shown in the bottom panel in Figure 3a. This behavior is indicated by the schematic of two collinear arrows pointing in the opposite directions.

Simultaneous activation of chemical influx from two adjacent side walls generates the convective flow shown in Figure 3b. The fluid motion resembles “dipolar” flow,^2^ which is controlled by a source and sink located at the bottom left and top right corners of the domain, respectively. This flow is directed along the diagonal in the central part of the domain (Figure 3b), but it becomes progressively more aligned with the side walls near the respective boundaries. The flow causes the posts to bend along the position-dependent streamlines (Figure 3b). The evolution of the average velocity field in the domain and the dependence of the glucose concentration on time and lateral distance are presented in the SI Appendix. Unlike the situation with two opposite active walls (which enabled two domains), the posts assemble into a single domain, where the local orientation of the posts gradually changes with the post’s position. The average post orientation can be represented by the diagonal arrow shown in Figure 3b.

Finally, for the case shown in Figure 3c, d-glucose is simultaneously introduced from all four side walls (see Movie S3 in the SI Appendix). The chemical influx is turned on for Δ*t*_on_ = 28 min and subsequently turned off for Δ*t*_off_ = 83 min. The rates of the influx at all the side walls are set to *R*_*i*_ = −*D***n***_i_* · ∇*C* = 7.01 × 10^–4^mol m^–2^ s^–1^, where **n**_*i*_ (1 ≤ *i* ≤ 4) is the normal vector to the respective four side walls. Here, the system generates four convective vortices that meet at the center of the domain. The 4-fold symmetry of the generated flow divides the entire array into four symmetric quadrants. In each quadrant, the position-dependent flow imposes hydrodynamic drag forces and bends the postslips along the local streamlines passing in the top half of the fluidic domain. Within each quadrant, the posts display a pattern that is like the dipolar motif shown in Figure 3b. Therefore, the orientation of the posts in the entire domain can be represented schematically by four diagonal arrows, where each arrow shows the orientation of the posts averaged over the respective quadrant.

**Figure 4:**
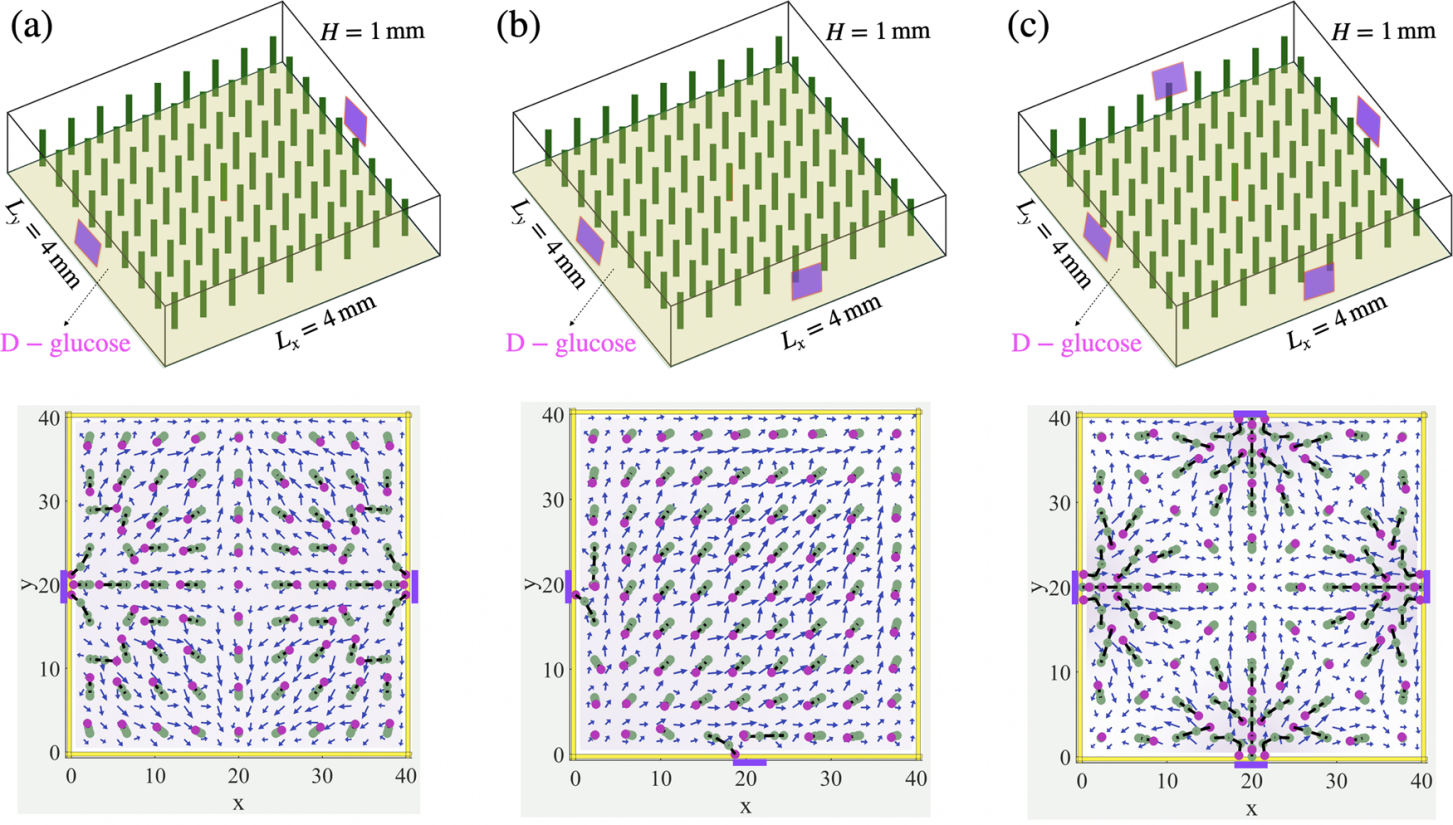
Convective flow generated by constant chemical influx from two and four patches in side walls of a microchamber. Top: schematic view of a fluid-filled chamber containing a 9 × 9 array of flexible posts, where reagent (released by a more dense d-glucose gel) enters the domain through (a) two patches in opposing side walls at *x* = 0 and *x* = 4 mm, (b) two patches in nearby side walls at *x* = 0 and *y* = 0, and (c) four patches in all the side walls; bottom: simulation result, showing the direction of the posts pointing toward the left and right patches in opposing side walls (a), two patches in lower nearby side walls (b), and four patches in all of the side walls (c). The chemical influx rate at all the patches in the side walls are set to *R* = 8.51 × 10^–4^ mol m^–2^ s^–1^.

In a relevant experimentally realized scenario,^34,35^ chemicals leak out of a portion of a reservoir (rather than the entire face of the wall). To model this situation, we introduce chemicals into the domain through small square patches localized on the side walls (see Figure 4). Each patch has an area that is 0.08 times the area of an entire side wall and acts as a source of chemicals (here, glucose) that produce the convective flow necessary to bend the passive posts.

Figure 4a–c shows a schematic of a fluid-filled chamber containing a 9 × 9 array of flexible posts, where the denser d-glucose enters the domain through: (a) two patches localized on the opposite side walls (at *x* = 0 and *x* = 4 mm); (b) two patches located on the adjacent side walls (at *x* = 0 and *y* = 0,); and (c) a patch on each of the four side walls. For cases (a) and (b), d-glucose is released simultaneously from both patches for a period Δ*t*_on_ = 14 min and then turned off for Δ*t*_off_ = 42 min. For case (c), d-glucose is released simultaneously from four side patches for a period Δ*t*_on_ = 28 min and then turned off for Δ*t*_off_ = 83 min. The average chemical concentrations and average velocity fields in the domain for these simulations are shown in Figure S5 in the SI.

The localized chemical sources generate position-dependent fluid flows that drag the tips of the posts along local streamlines in the top half of the fluidic domain. Although the local tilting of the post depends on its position, the orientations averaged over the appropriate subdomain provide information about the net post orientation. In the case shown in Figure 4a, the post orientations averaged across the respective halves of the domain can be schematically shown by two collinear arrows pointing in opposite directions. The two symmetric subdomains are separated by a vertical region, where all of the posts assume straight configurations due to their equal distances from the two chemical sources.

In the situation shown in Figure 4b, the post orientations averaged across the entire domain can be presented by the diagonal arrow shown in Figure 4b. In the case of four chemical sources (Figure 4c), the entire domain is separated into four subdomains by the single-file line of posts along the vertical and horizontal axes that pass through the center of the chamber. All of the posts along the latter dividing lines assume vertical configurations due to their symmetric positions with respect to the four sources. The post orientations averaged over the four quadrants can be presented as four diagonal arrows (similar to those shown in Figure 3c).

### III.B Effects of Solutal Buoyancy Produced by Chemical Reactions: Diffusion–Reaction–Convection

#### 1 One-Stage Reaction

**Figure 5:**
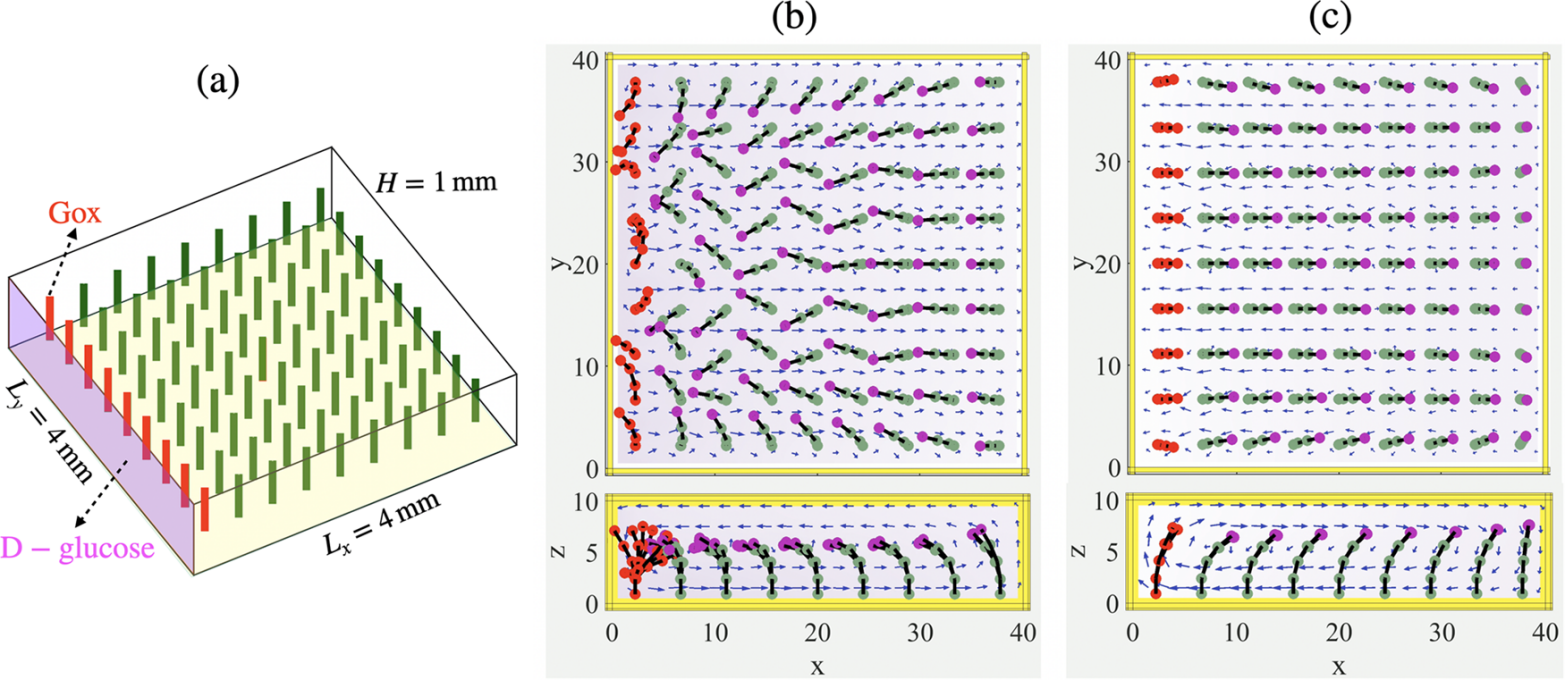
Convective and one-stage reactive flows generated by constant chemical influx from one side wall of a microchamber. (a) Schematic view of a fluid-filled chamber containing 9 GOx-coated posts and 72 passive posts in an array of 9 × 9 flexible posts, where reagent (released by a more dense d-glucose gel) enters the domain through the left side wall at *x* = 0; reagent enters the domain for 42 min and then turned off. (b, c) Snapshot of the simulation results of the time-dependent motion of posts within the first influx time (b), where the posts bend toward the left side wall, and after turning off the chemical influx (c), where the posts change their direction toward the opposing side wall; see Movie S4 in the SI Appendix. The chemical influx rate at the left side wall is set to *R*_1_ = −*D*∂*C*/∂*x*(*x*=0) = 1.26 × 10^–2^ mol m^–2^ s^–1^, and the reaction rate at the surface of the GOx-coated posts is *r*_m_^gox^ = 7.88 × 10^–6^ mol m^–2^ s^–1^.

As noted above, the density gradients (and resulting solutal buoyancy effects) in the fluidic system can be introduced in two ways: (1) inherent density variations produced by nonreactive chemicals in water and (2) density differences between reactants and products that participate in the chemical reaction. Both processes can occur simultaneously in the same chamber, permitting significant control over the spatiotemporal behavior and pattern formation in the array of elastic posts. The first mechanism can be realized by introducing d-glucose from the left side wall (*x* = 0) in a chamber that contains a 9 × 9 array of elastic posts. To employ the second mechanism, we assume that the first nine posts near the left side wall are coated with glucose oxidase (GOx) (Figure 5a).

In the first stage, the glucose-rich solution that emanates from the left side wall moves downward due to gravity. At the same time, the less dense glucose-poor solution at the right boundary rises upward and moves along the top plane back to the left boundary, as in Figure 1a. This convective, circular flow causes the flexible posts to bend toward the left side wall, as shown in Figure 1b. At this stage, the fluid velocity is sufficiently large, and thus, d-glucose is carried by the flow toward the nine active posts. Note that the convective flow generated by this constant chemical influx is stronger than the buoyancy-driven flows that result from chemical reactions since there is not enough d-glucose in the domain to generate comparable effects. After the injection of d-glucose from the left is turned off, the first mechanism behind the convective flow stops, and there is sufficient time for glucose in the solution to react with the GOx-coated posts (colored red in Figure 5a) and induce buoyancy flow due to the chemical reaction. Specifically, the reaction at the surface of the active posts decomposes d-glucose to gluconic acid and hydrogen peroxide (H_2_O_2_):
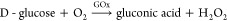
6

Since the products are less dense than the reactant (d-glucose), the GOx-coated posts generate inward flow so that the nearby passive posts bend away from the GOx-coated posts, as seen in Figure 5c. This bending involves the reorientation of the posts from pointing to the left to pointing to the right and resembles a wave-like motion, which is triggered by the chemical influx and propagates back and forth across the domain. Note that we ignore the presence of oxygen in the simulations because the solutal buoyancy expansion coefficient of oxygen is approximately an order of magnitude smaller than that for hydrogen peroxide.

In the simulations, dynamic reorientation of the posts in the radial directions is achieved by coating all the posts located adjacent the four side walls with GOx (Figure S6a). In this scenario, glucose enters the domain through all the side walls for 84 min and produces a radially oriented convective flow that forces all the posts to bend radially away from the center of the domain (Figure S6b). When the chemical influx is turned off, the GOx reaction at the surface of the active posts starts to control the flow patterns and switches the direction of the convective vortices. The resulting outward flow drags the neighboring passive posts and bends them toward the center of the domain(Figure S6c).

#### 2 Two-Stage Reaction

**Figure 6:**
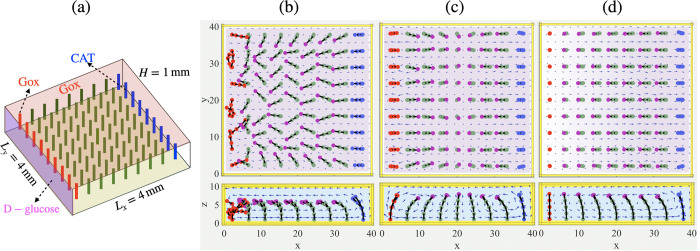
Convective and two-stage reactive cascade flows generated by constant chemical influx from one side wall of a microchamber. (a) Schematic view of a fluid-filled chamber containing 9 GOx-coated posts (colored in red) near the left side wall, 9 CAT-coated posts (colored in blue) near the right side wall and 63 passive posts (green) in an array of 9 × 9 flexible posts. Reagent (released by a more dense d-glucose gel) enters the domain through the left side wall at *x* = 0. The top wall was also coated with GOx to increase the effective reaction rate. The reagent enters the domain for 84 min and then turned off; (b–d): Snapshot of the simulation results of the time-dependent motion of posts within the first influx time (b), where the posts bend toward the left side wall; after turning off the chemical influx, with dominating flow by GOx reaction (c), where the posts near GOx-coated posts change their direction toward the right side wall; and with dominating flow by CAT reaction (d), where the posts bend toward the left side wall; see Movie S6 in the SI Appendix. The chemical influx rate at the left side wall is set to *R*_1_ = −*D*∂*C*/∂*x*(*x*=0) = 2.52 × 10^–2^ mol m^–2^ s^–1^, and the reaction rates are *r*_m,post_^gox^ = 7.88 × 10^–6^ mol m^–2^ s^–1^, *r*_m,post_^cat^ = 1.62 × 10^–4^ mol m^–2^ s^–1^, and *r*_m,(topwall)_^gox^ = 3.67 × 10^–7^ mol m^–2^ s^–1^.

Additional control over time-dependent patterns of posts could be induced with cascade reactions (that follow the chemical influx stage), where the products of the first reactions become reactants for the second. To illustrate this scenario, we consider a time-dependent behavior in a square array of 9 × 9 elastic posts confined between two rows of active posts, where the most left row (nine posts shown in red in Figure 6) is coated with glucose oxidase (GOx) and the most right posts (nine posts shown in blue) are coated with catalase (CAT). With the injection of d-glucose (through the left wall), the system undergoes a two-step cascade reaction:
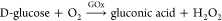
7
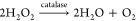
8

Namely, the GOx-coated posts catalyze the consumption of d-glucose to produce H_2_O_2_, which is consumed in the second reaction by the CAT-coated posts to produce water and oxygen. Since the rate of reaction at the first stage is an order of magnitude slower than the reaction rate at the second stage, to increase the effective reaction rate (which is proportional to the area coated with enzyme^31,47,48^) of the first reaction, we also coat the top walls of the chamber with GOx.

The dynamic behavior of the system is initiated by d-glucose, which enters the domain through the left side wall for the first Δ*t*_on_ = 84 min, and then this influx is turned off. At the first stage (before the glucose influx is turned off), the denser glucose at the left side wall moves downward generating a counterclockwise circulating flow that drags the flexible posts and bends their tips toward the left side wall (see Figure 6b).

Note that at the first stage (i.e., before we turn off the chemical influx from the left side wall), GOx-coated posts catalyze the consumption of d-glucose and the production of hydrogen peroxide that serves as a fuel for the second reaction on the surface of CAT-coated posts. The convective flow generated by the constant chemical influx is stronger than the buoyancy-driven flows produced by the first stage of the chemical reactions because there are not enough reactants in the domain to generate comparable effects. Nevertheless, there is a competition between influx-generated convection and buoyancy-driven flow produced near the GOx-coated posts, as evidenced by the unstable oscillatory/chaotic behavior of the red posts (Figure 6b). The production (consumption) of chemicals is controlled by the area of the enzyme-coated surfaces, *S*, the magnitude of the turnover rate *k*_*e*_ of each enzyme molecule, and the areal concentration of the molecules, [*E*]. Increasing any of these parameters increases the production (consumption) of the chemicals in the chamber. Using this fact, we coat the top wall with GOx (with a lower reaction rate than the GOx-coated posts) to produce a sufficient amount of hydrogen peroxide for the reactions on CAT-coated posts.

After we turned off the glucose influx from the left side wall, the reactions on the GOx-coated posts became dominant; because the products are lighter than the reactants, this reaction generates inward flow at the bottom and causes a clockwise circulatory flow that bends the nearby passive posts away from the GOx-coated posts (Figure 6c). The reduction and ultimate consumption of d-glucose stops the reaction on the GOx-coated posts, and since there is enough hydrogen peroxide in the domain, the second reaction on the surface of CAT-coated posts starts to dominate; the counterclockwise circulatory fluid flow drives the nearby passive posts to bend horizontally away from the right side wall (Figure 6d). This dynamic reorientation can be viewed as a wave, which is introduced by the chemical influx and transmitted along the domain in the forward and backward directions.

The system can be adjusted to display dynamic reorientation of the posts in the radial direction. This scenario can be realized by placing the GOx-coated posts along all side walls and positioning CAT-coated posts at the center of the domain, as shown in SI Figure S7. We consider a fluid-filled chamber with a square array of 9 × 9 elastic posts tethered to the bottom wall; 32 posts adjacent to the side walls are coated with GOx and 9 posts in the middle of the domain are coated with CAT. The combined effects of the injection of d-glucose through all the side walls and the two-stage reaction generate a radial buoyancy-driven flow that drags the posts in the radial direction. The sequence of dynamic changes in the post orientation is shown in Figure S7b–d.

## IV Conclusions

We showed that for an aqueous solution, the addition of reagents having a different mass-to-volume ratio than water spontaneously generates mechanical forces that regulate the shape and organization of microscale objects immersed in the solution. In previous studies,^2^ we showed that if an enzymatic reaction produced products with a different volume than the reactants, the local density gradients generated solutal buoyancy forces that spontaneously performed the mechanical work to propel fluids and controllably reconfigure immersed 2D and 3D materials, including the tethered microposts. The findings presented here show that this form of mechanical work can occur autonomously even without chemical reactions. Numerous reagents can be used to trigger motion and shape change in water. In the absence of tethered microposts, fluid motion due to solutal buoyancy can be harnessed to controllably transport microparticles to specific locations in the chamber.^31^ These results have broad implications for significantly augmenting the range of chemicals used to achieve autonomous functionality in fluidic devices.

Systems involving diffusion–reaction processes are well-known to drive pattern formation in solution and have been used to tailor the spatial and temporal evolution of complex fluids.^49^ The diffusion–reaction–convection mechanisms proved to be important in the processes of transport and control with the microscale objects (like particles and sheets^34,35^) by the use of chemical catalytic pumps. Our findings show that the latter convective processes can be produced by the nonreactive chemicals that broaden the choice of possible reactants and provide additional tools for regulating the system’s nonequilibrium dynamics.

In a system with a given arrangement of active and passive posts, turning the chemical influx on and off leads to the formation of propagating waves that drive the posts to exhibit biomimetic cilia-like motion. The time period of the on and off intervals can be used to manipulate the waves’ properties and the posts’ cilial dynamics. We also demonstrated how staging the sequence of release from the different side walls provided another means of varying the propagation of waves in the chamber. Additionally, the introduction of cascade reactions can be used to dynamically shift the direction of the wave propagation. Finally, the number and placement of the tethered enzyme-coated, active posts, and passive posts within the array constitute an important variable for directing the structure formation and dynamic behavior in the fluidic chambers.

With and without posts, the above systems innately perform biomimetic chemo-mechanical transduction, utilizing chemical input to instigate and sustain mechanical action. It remains a significant challenge to imbue synthetic systems with innate chemo-mechanical transduction and thus fabricate objects that exhibit the level of autonomy needed to create self-operating soft robots. The system described here offers one means of moving closer to addressing the latter challenge. Notably, living systems contain a myriad of chemical reactions in confined fluids. The findings indicate that intrinsic hydrodynamics and fluid–structure interactions cannot be ignored in characterizing fluid flow and fluid–structure interactions in biology.

## Supporting Information Available

Details of elastic sheet model, simulation parameters, and supplementary figures (PDF)
(Movie S1) Convective flow generated by sequential release of constant chemical influx from all the four side walls of a microchamber (MP4)
(Movie S2) Convective flow generated by constant chemical influx from two opposing and adjacent side walls and simultaneous release form all four side walls of a microchamber (MP4)
(Movie S3) Convective flow generated by constant chemical influx from two patches in opposing and adjacent side walls and simultaneous chemical release from a patch on each of the four side walls of a microchamber (MP4)
(Movie S4) Convective flows generated by constant chemical influx from one side wall of a microchamber and one-stage reaction from a line of 9 GOx-coated posts near a side wall (MP4)
(Movie S5) Convective flows generated by constant chemical influx from one side wall of a microchamber and two-stage reactions from a line of 9 GOx-coated posts and a line of CAT-coated posts near the left and right side walls, respectively (MP4)
(Movie S6) Convective flow generated by sequential release of constant chemical influx from all the four side walls of a microchamber, transporting microparticles (MP4)
(Movie S7) Convective flow generated by sequential release of constant chemical influxes from two opposing side walls transporting nonbuoyant microparticles (MP4)
(Movie S8) Convective flow generated by sequential release of constant chemical influx from two adjacent side walls transporting microparticles (MP4)
(Movie S9) Convective flow generated by simultaneous release of constant chemical influx from all the side walls transporting microparticles (MP4)
(Movie S10) Convective and one-stage reactive flows generated by simultaneous release of constant chemical influx from all the side walls of microchamber (MP4)
(Movie S11) Convective and two-stage reactive flows generated by simultaneous release of constant chemical influx from all the side walls of microchamber (MP4)
