# Supplementary material for: Controlling the Dynamic Behavior of Microposts in Solution via Diffusion–Convection
Source: Langmuir. 2025 Mar 5;41(10):6633–43. doi: 10.1021/acs.langmuir.4c04567 (PMC11924236; doi:10.1021/acs.langmuir.4c04567)
Supplement: Supplementary file 1 — la4c04567_si_001.pdf [file la4c04567_si_001.pdf]

Supplementary Information for

Controlling the dynamic behavior of microposts in solution via diffusion-convection

Moslem Moradi [a] *, Oleg E. Shklyaev* [a]*, and Anna C. Balazs* [a,*]

[a] Department of Chemical Engineering, University of Pittsburgh, Pittsburgh, PA, 15261
* Email: balazs@pitt.edu

I. Model for elastic post

An elastic post is modeled as a one-dimensional network of nodes connected by elastic bonds. The elastic energy of the connecting bonds is based on the Kirchhoff's rod model and can be written as[1]:

$$E = \frac{1}{2}\int \left[b_1\left(\mathbf{D_1}\cdot\frac{\mathrm{d}\mathbf{r}}{\mathrm{d}s}\right)^2 + b_2\left(\mathbf{D_2}\cdot\frac{\mathrm{d}\mathbf{r}}{\mathrm{d}s}\right)^2 + b_3\left(\mathbf{D_3}\cdot\frac{\mathrm{d}\mathbf{r}}{\mathrm{d}s}-1\right)^2 \right]\mathrm{d}s$$

$$+\frac{1}{2}\int \left[a_1\left(\frac{\mathrm{d}\mathbf{D_2}}{\mathrm{d}s}\cdot\mathbf{D_3}\right)^2 + a_2\left(\frac{\mathrm{d}\mathbf{D_3}}{\mathrm{d}s}\cdot\mathbf{D_1}\right)^2 + a_3\left(\frac{\mathrm{d}\mathbf{D_1}}{\mathrm{d}s}\cdot\mathbf{D_2}\right)^2 \right]\mathrm{d}s\,. \qquad \text{(S1)}$$

Here $a_1 = a_2 = \kappa_b$ is the bending modulus and a_3 is the twist modulus of an isotropic rod. Also, $b_1 = b_2$ is the shear force constant and $b_3 = \kappa_s$ is stretching modulus of the rod. $\mathbf{D_1}$, $\mathbf{D_2}$ and $\mathbf{D_3}$ are are the unit orthonormal directors at each node of the post and s is the arc length along the elastic bonds. The respective dimensions of κ_s and κ_b are force and force$\times L^2$, where L is the length of the rod. The stretching and bending modulus of the rod allow us to characterize the elastic forces experienced by the posts' nodes.

The steric repulsion forces on a post, $\mathbf{F}_s$, is the sum of the repulsive force between any two nodes in the post ("node-node" (nn)) and the repulsive force between the posts' nodes and the nodes that make up the six confining walls. The repulsion force between node i and j is given as $\mathbf{F}^{nn}(\mathbf{r}_i - \mathbf{r}_j) = -\nabla U(r)$, where $U(r)$ is the Morse potential:

$$U(r) = \begin{cases} \varepsilon\left(1-\exp[-\omega(r-r_0)]\right)^2, & r < r_0 \\ 0, & r \ge r_0. \end{cases} \qquad \text{(S2)}$$

Here, $r = |\mathbf{r}_i - \mathbf{r}_j|$ is the distance between the position of the i-th node and the repelling object $\mathbf{r}_j$ (other nodes or wall). Also, ε is the strength of the potential, ω is it's width, and r_0 is the equilibrium (and cutoff) distance. The value of r_0 is taken as $1.3\Delta x$, where the lattice Boltzmann unit Δx corresponds to $100\,\mu\mathrm{m}$. The simulations are run for over 8×10^6 iterations, corresponding to 3.7 hours of physical time.

II. Parameters characterizing the mechanical properties of the posts

For a tethered flexible cantilever[2], the vertical displacement of the cantilever, *w(x)*, when a force F is applied to the free end is equal to $w(x) = \frac{\mathrm{F}}{6EI}(3Lx^2 - x^3)$, where E is the Young's modulus and I is the second moment of the areal cross section. Figure S1(b) shows the final displacements of the nodes from both theory and simulation for two different values of the bending stiffness κ_b and Fig. S1(c) shows the deflection of the top node as a function of time for the same two posts.

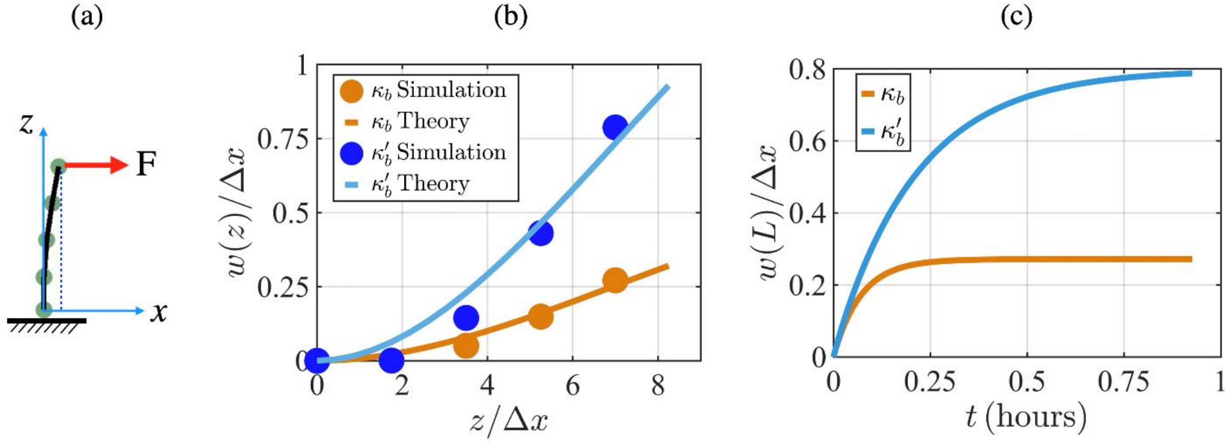

Figure S1 | Estimating bending modulus of the elastic post. (a) Schematic of the post deflection under the action of a constant force. (b) Deflection of the five nodes of the post as a function of vertical position of the nodes from simulation and with the analytical expression for two bending stiffness of the post. (c) Deflection of the top node as a function of time for two bending stiffness of the post. The applied force at the end of the post is $\mathrm{F} = 10^{-14}\,\mathrm{N}$.

We use the vertical displacement of the end of the post for a given load to compute the flexural rigidity $\mathrm{B} := EI = \frac{\mathrm{F}L^3}{3w(L)}$ of the post. We find $\mathrm{B} = 0.04\,p\mathrm{Nmm}^2$ and $\mathrm{B}' = 0.014\,p\mathrm{Nmm}^2$, for the two posts, corresponding to bending stiffness of $\kappa_b = 0.17\,p\mathrm{Nmm}^2$ and $\kappa_b' = 0.057\,p\mathrm{Nmm}^2$, respectively. Experiments[3-5] on flexible materials that are characterized by the same length scale as in the simulations show values that fall in this range, suggesting candidate materials that can be used in experiments to realize the modeled system.

III. Physical values corresponding to characteristics the solutes and enzymes

Table S1 Physical properties of solutes

Chemicals	Density ρ (g/cc)	Diffusivity D (m^2/s)	Molar mass m_m (g/mol)	Expansion coefficient β (M^{-1})
H_2O	1.00	-	18.015	-
H_2O_2	1.45	1.43×10^{-9}	34.015	0.01056
$C_6H_{12}O_6$	1.54	0.67×10^{-9}	180.16	0.063
$C_6H_{12}O_7$	1.24	0.9×10^{-9}	196.16	0.038

Table S2 Parameters for enzymes

Enzyme	k_e (s^{-1})	K_M (M)	$[E]$ (M)
catalase (CAT)	2.1×10^5	0.093	0.5×10^{-6}
GOx	1840	0.03	2.8×10^{-6}

The values for densities and diffusivities are taken from established references [6-8]. The values for the physical characteristics of the enzymes were also taken from established references[9-12].

IV. Supplementary figures

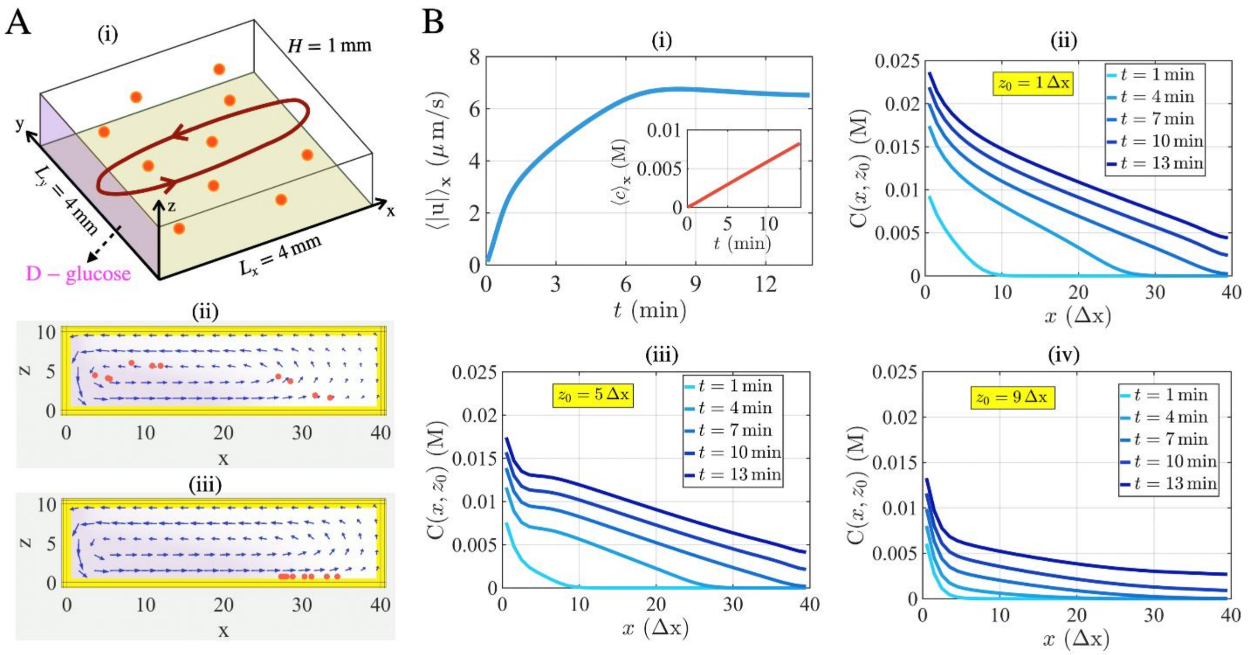

Figure 1 | Convective flow generated by a constant chemical influx from left side wall of a micro-chamber. A(i) Schematic view of a fluid-filled chamber (of dimensions $40\Delta x \times 40\Delta x \times 10\Delta x$ where $\Delta x = 0.1\,\mathrm{mm}$ is the lattice Boltzmann unit) containing passive tracer particles, where released reagents (D-glucose) enters the domain through the left side wall at $x = 0$. A(ii)-(iii) Side view of the flow generated by the influx of chemicals through the left side wall where the particles (tracers) have the same density as the fluid A(ii), and where the particles are denser than the fluid A(iii). B(i) Average velocity field and glucose concentration (*inset*) in the domain as a function of time. B(ii)-(iv) Concentration field as a function of lateral distance from the left side wall, x, at different times for three different heights: (ii) $z = 0.1\,\mathrm{mm}$, (iii) $z = 0.5\,\mathrm{mm}$ and (iv) $z = 9\,\mathrm{mm}$. The chemical influx rate at the left sidewall is set to $R_1 = -D\partial C / \partial x\,(x = 0) = 3.14 \times 10^{-3}\,\mathrm{mol\,m^{-2}s^{-1}}$.

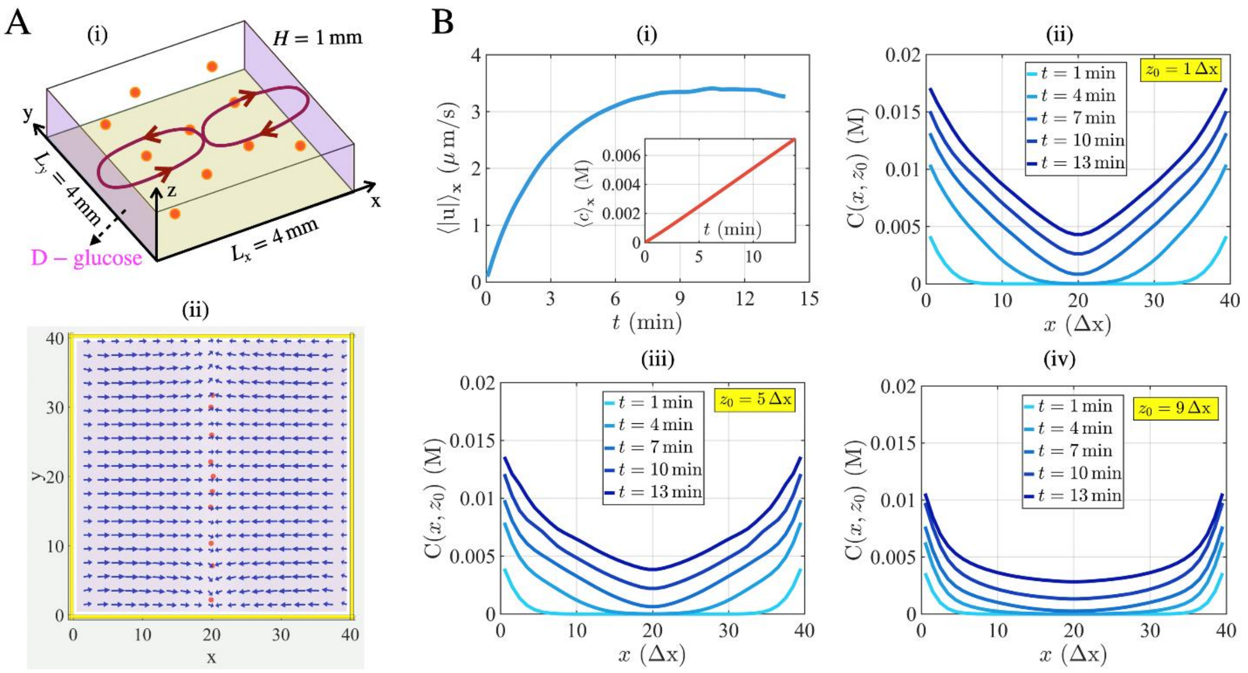

Figure S2 | Convective flow generated by constant chemical influx from two opposing side walls of a microchamber. (A): (i) Schematic view of a fluid-filled chamber containing passive tracer particles, where D-glucose enters the domain through two *opposing* sidewalls at $x = 0$ and $x = 4\,\mathrm{mm}$; (ii) Simulation results showing the transport of dense particles along the center line of the domain. The blue arrows show the direction of the flow at the bottom of the chamber. (B): (i): average velocity field and glucose concentration (*inset*) in the domain. (ii)-(iv): concentration field as a function of horizontal axis, x, at different times (light to dark blue) for three different heights: (ii) $z = 0.1\,\mathrm{mm}$, (iii) $z = 0.5\,\mathrm{mm}$ and (iv) $z = 9\,\mathrm{mm}$ ($\Delta x = 0.1\,\mathrm{mm}$). The chemical influx rate at the two opposing side walls, i.e, $R_1 = -D\partial C / \partial x\,(x = 0)$ and $R_2 = D\partial C / \partial x\,(x = 4)$, are set to $R_1 = R_2 = 1.57 \times 10^{-3}\,\mathrm{mol m^{-2} s^{-1}}$.

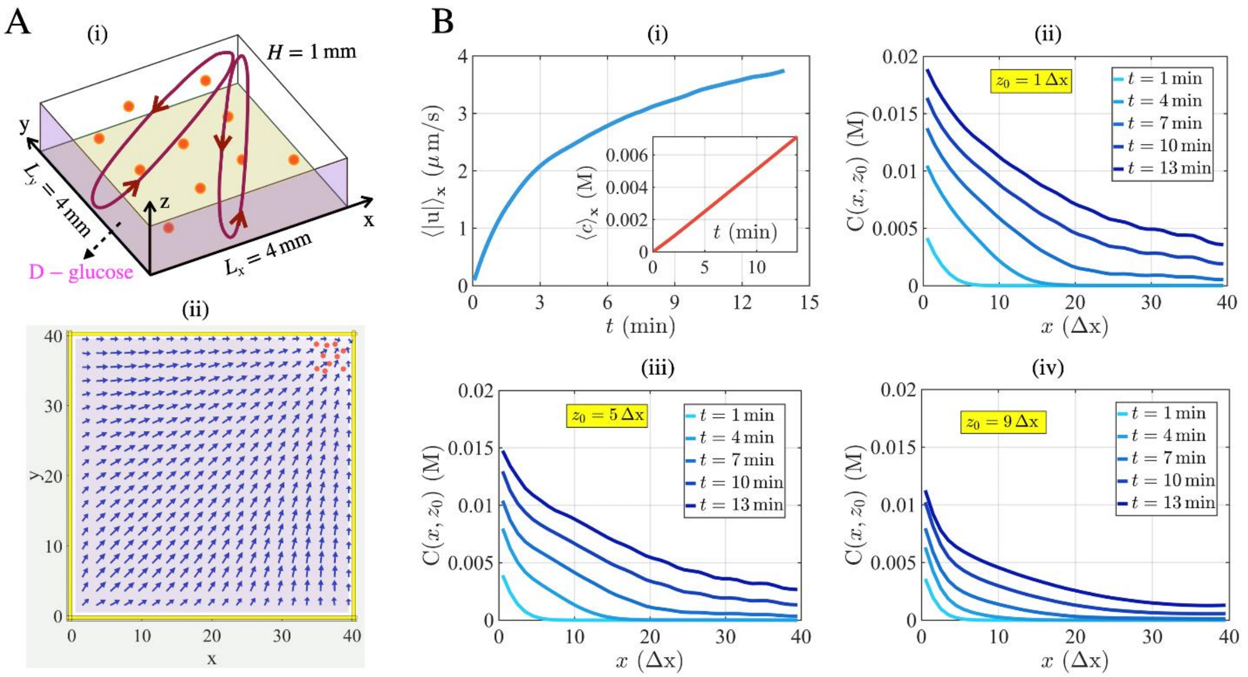

Figure S3 | Convective flow generated by constant chemical influx from two nearby side walls of a microchmaber. (A): (i) Schematic view of a fluid-filled chamber containing passive tracer particles, where D-glucose gel enters the domain through *two nearby* sidewalls at $x = 0$ and $y = 0$; (ii) Simulation results showing the transport of dense particles along the diagonal line and toward corner of the domain. Glucose influx is switched on for 14 minutes. The blue arrows show the direction of the flow at the bottom of the chamber. (B): (i): average velocity field and glucose concentration (*inset*) in the domain. (ii)-(iv): concentration field as a function of horizontal axis, x , at different times (light to dark blue) for three different heights: (ii) $z = 0.1\,\mathrm{mm}$, (iii) $z = 0.5\,\mathrm{mm}$ and (iv) $z = 9\,\mathrm{mm}$ ($\Delta x = 0.1\,\mathrm{mm}$). The chemical influxes rate at the two nearby sidewalls, i.e, $R_1 = -D\partial C / \partial x\,(x = 0)$ and $R_2 = -D\partial C / \partial y\,(y = 0)$, are set to $R_1 = R_2 = 1.57 \times 10^{-3}\,\mathrm{mol m^{-2} s^{-1}}$.

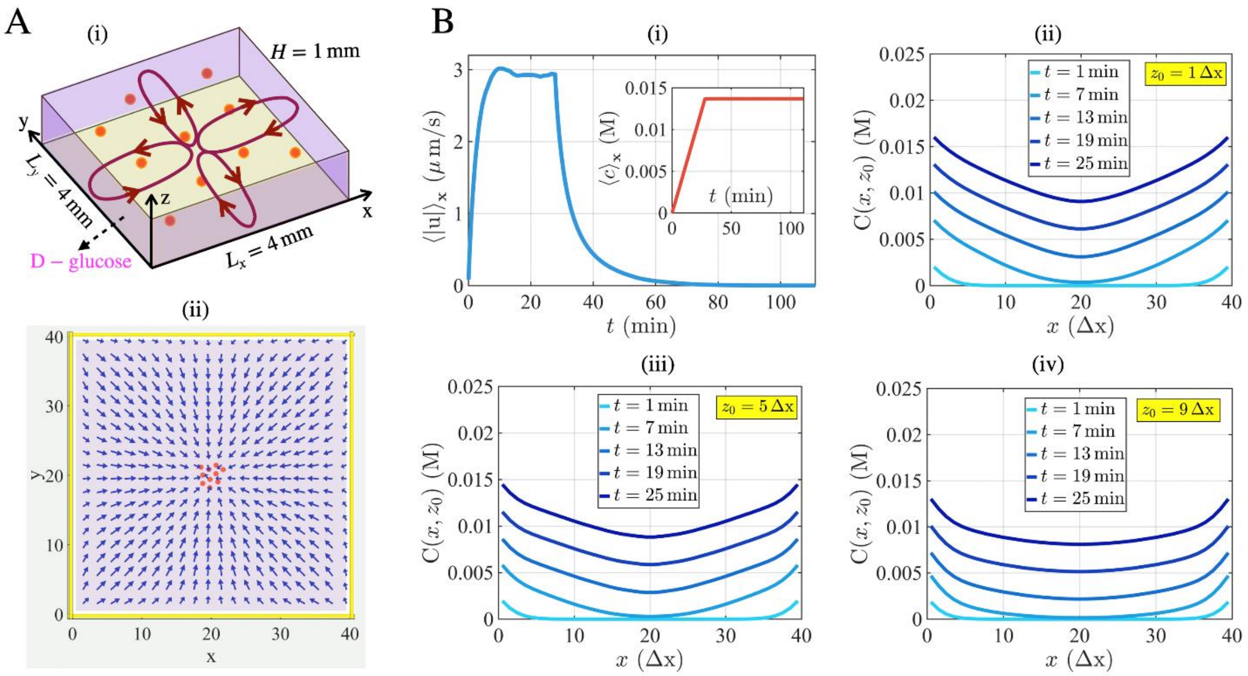

Figure S4 | Convective flow generated by constant chemical influx from all side walls of a microchamber. (A): (i) Schematic view of a fluid-filled chamber containing passive tracer particles, where D-glucose enters the domain through *all* side walls at $x = 0, 4$ mm and $y = 0, 4$ mm ; (ii) Simulation result showing the transport of dense particles toward the center of the domain. Glucose influx is switched on for 28 minutes, and then is switched off. The blue arrows show the direction of the flow at the bottom of the chamber. (B): (i): average velocity field and glucose concentration (*inset*) in the domain. (ii)-(iv): concentration field as a function of horizontal axis, x , at different times (light to dark blue) for three different heights: (ii) $z = 0.1$ mm , (iii) $z = 0.5$ mm and (iv) $z = 9$ mm ($\Delta x = 0.1$ mm). The chemical influx rate at all the side walls are set to $R_i = -D\mathbf{n}_i \cdot \nabla C = 7.01 \times 10^{-4} \mathrm{mol m^{-2} s^{-1}}$, where $\mathbf{n}_i, (1 \le i \le 4)$ is the normal vector to respective four sidewalls.

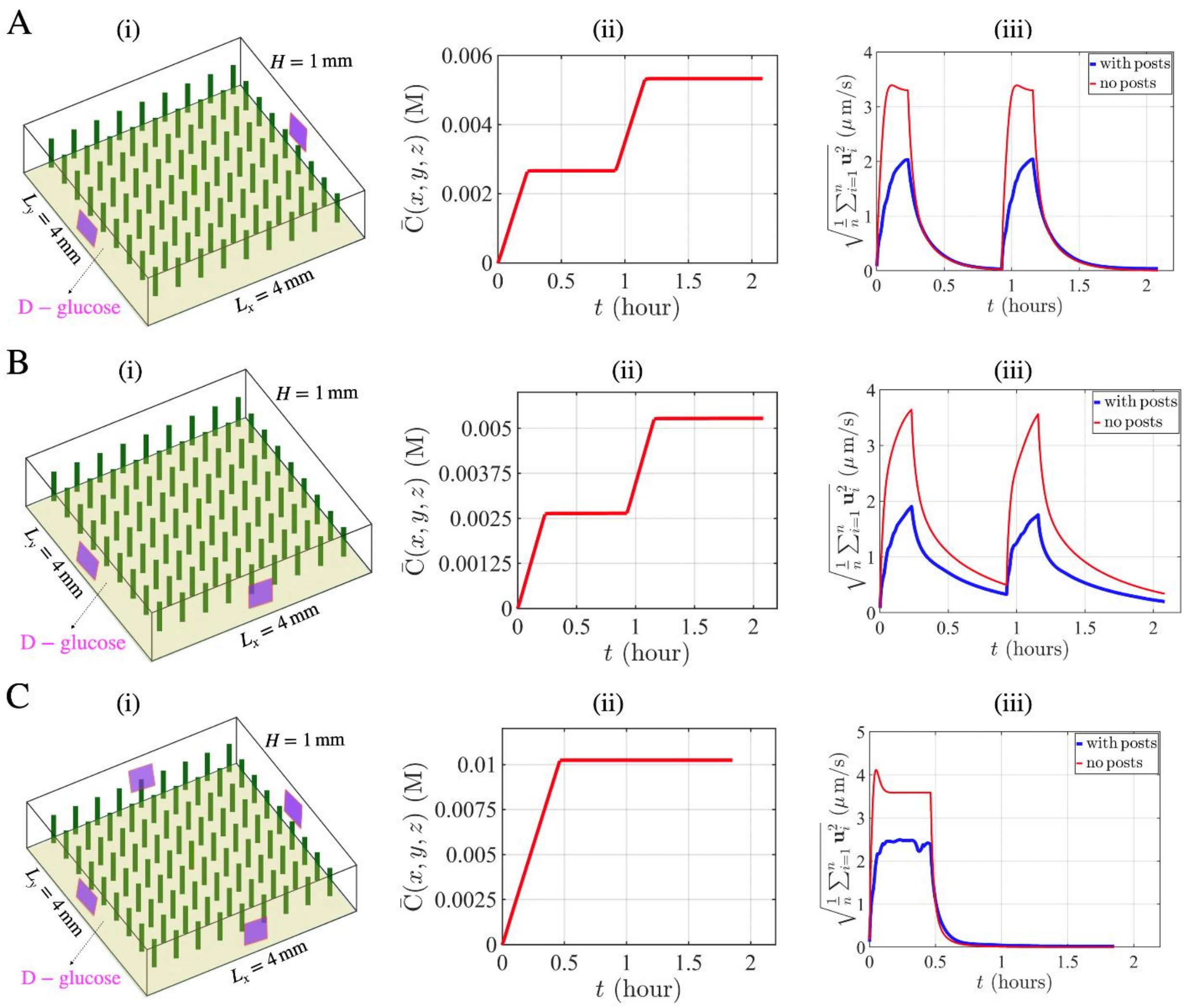

Figure S5 | Convective flow generated by constant chemical influx from two and four patches on side walls of a microchamber. Schematic view of a fluid-filled chamber containing a 9×9 array of flexible posts, where D-glucose enters the domain through: A(i) two patches in opposing side walls at $x=0$ and $x=4\text{mm}$, B(i) two nearby patches in side walls at $x=0$ and $y=0$, and C(i) four patches in all the side walls. D-glucose enters the domain from two patches in A and B for 14min and then is switched off for 42min. For case C, D-glucose enters the domain from a patch on each of the four of the side walls for 28min and then is switched off for 83min. In middle panels, we plot the average glucose concentration and the average velocity field in the domain (with and without posts) as a function of time (see Movie S8 in *SI Appendix*). The chemical influx rate at all the patches in the side walls are set to $R_i = -D\mathbf{n}_i\cdot\nabla C = 8.51\times10^{-4}\,\text{molm}^{-2}\text{s}^{-1}$, where $\mathbf{n}_i, (1\le i\le 4)$ is the normal vector to respective four sidewalls.

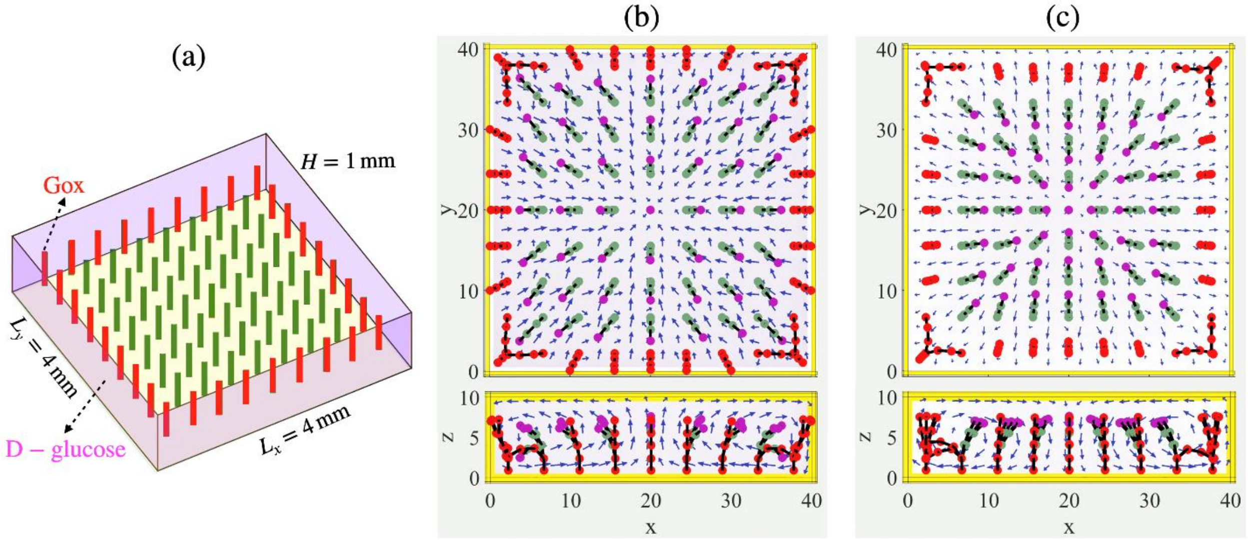

Figure S6 | Convective flows generated of 1-stage reaction on the active posts and constant chemical influx from all side walls of a microchamber. (a) Schematic of a fluid-filled chamber containing 32 GOx-coated posts and 49 passive posts in the 9×9 array. The reactant enters the domain uniformly from all four walls; the influx remains on for 84 min. (b) Snapshot of the time-dependent motion of posts due to the initial influx of reactants, where the posts bend radially away from the center. (c) After the chemical influx is shut off, the posts change direction and bend radially toward the center; see Movie S5 in *SI Appendix*. The chemical influx rate at all of the sidewall is set to $R_i = -D\mathbf{n}_i \cdot \nabla C = 1.57 \times 10^{-3}\,\mathrm{mol m^{-2} s^{-1}}$, where $\mathbf{n}_i, (1 \le i \le 4)$ is the normal vector with respect to a side wall *i*. The reaction rate at the surface of the GOx-coated posts is $r_\mathrm{m}^\mathrm{gox} = 7.88 \times 10^{-6}\,\mathrm{mol m^{-2} s^{-1}}$.

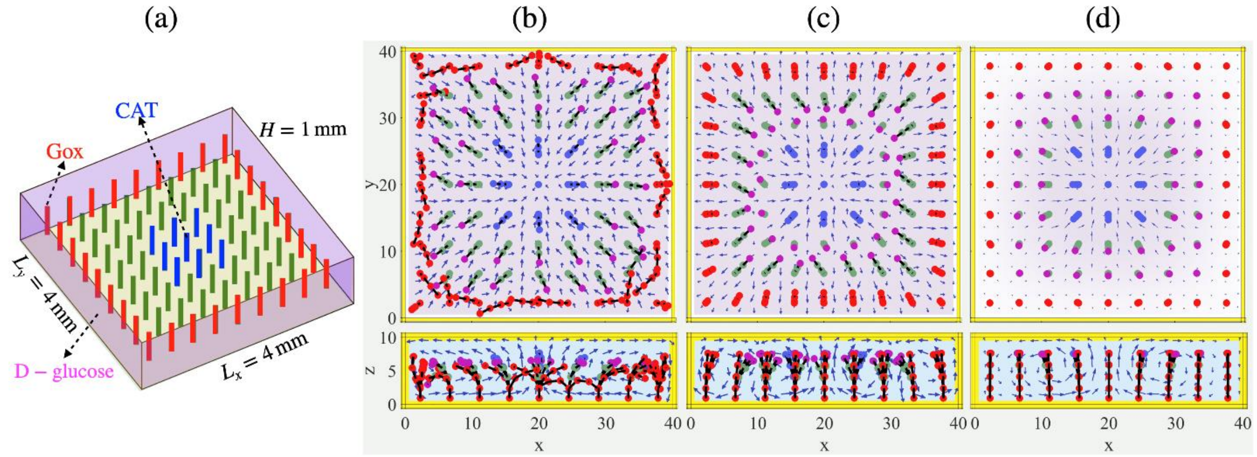

Figure S7 | Convective flow generated by 2-stage cascade reaction and constant chemical influx from all four sidewalls of a microchamber. (a) Chamber contains 32 GOx-coated posts near the sidewalls (colored in red), 9 CAT-coated posts in the middle (blue) and 40 passive posts (green) in the 9×9 array of flexible posts. D-glucose enters the domain through all four sidewalls for 84 minutes and then the influx is switched off. (b) The initial influx of D-glucose causes the posts to bend radially away from the center. (c) After switching off the chemical influx, the reaction between D-glucose and the GOx-coated posts generates H_2O_2 and outward flow that causes the posts to bend toward the center. (d) The inward flow generates by the CAT reaction drives all the posts to bend radially away from the center; see Movie S7 in *SI Appendix*. The chemical influx rate at all of the sidewall are set to $R_i = -D\mathbf{n}_i \cdot \nabla C = 1.57 \times 10^{-3} \text{molm}^{-2}\text{s}^{-1}$, where $\mathbf{n}_i, (1 \le i \le 4)$ is the normal vector to the ith side wall. The reaction rates are $r_{\text{m,post}}^{\text{gox}} = 7.88 \times 10^{-6}\ \text{molm}^{-2}\text{s}^{-1}$, $r_{\text{m, post}}^{\text{cat}} = 1.62 \times 10^{-4}\ \text{molm}^{-2}\text{s}^{-1}$.

V. Supplementary movies

Supplementary Movie S1: Convective flow generated by sequential release of constant chemical influx from all the four side walls of a micro-chamber.

Supplementary Movie S2: Convective flow generated by constant chemical influx from two opposing and adjacent side walls and simultaneous release form all four side walls of a micro-chamber.

Supplementary Movie S3: Convective flow generated by constant chemical influx from two patches in opposing and adjacent side walls and simultaneous chemical release from a patch on each of the four side walls of a micro-chamber.

Supplementary Movie S4: Convective flows generated by constant chemical influx from one side wall of a micro-chamber and 1-stage reaction from a line of 9 GOx-coated posts near a side wall.

Supplementary Movie S5: Convective flows generated by constant chemical influx from one side wall of a micro-chamber and 2-stage reactions from a line of 9 GOx-coated posts and a line of CAT-coated posts near the left and right side walls, respectively.

Supplementary Movie S6: Convective flow generated by sequential release of constant chemical influx from all the four side walls of a micro-chamber, transporting microparticles.

Supplementary Movie S7: Convective flow generated by sequential release of constant chemical influxes from two opposing side walls transporting non-buoyant microparticles.

Supplementary Movie S8: Convective flow generated by sequential release of constant chemical influx from two adjacent side walls transporting microparticles.

Supplementary Movie S9: Convective flow generated by simultaneous release of constant chemical influx from all the side walls transporting microparticles.

Supplementary Movie S10: Convective and 1-stage reactive flows generated by simultaneous release of constant chemical influx from all the side walls of micro-chamber.

Supplementary Movie S11: Convective and 2-stage reactive flows generated by simultaneous release of constant chemical influx from all the side walls of micro-chamber.

References

1 Lim, S., Ferent, A., Wang, X. S. & Peskin, C. S. Dynamics of a Closed Rod with Twist and Bend in Fluid. *SIAM Journal on Scientific Computing* **31**, 273-302 (2008). https://doi.org:10.1137/070699780

2 Manna, R. K., Shklyaev, O. E. & Balazs, A. C. Chemical pumps and flexible sheets spontaneously form self-regulating oscillators in solution. *Proc Natl Acad Sci U S A* **118** (2021). https://doi.org:10.1073/pnas.2022987118

3 Lin, Y. *et al.* Ultrathin cross-linked nanoparticle membranes. *Journal of the American Chemical Society* **125**, 12690-12691 (2003).

4 Lee, D. Y. *et al.* Macroscopic nanoparticle ribbons and fabrics. *Adv. Mater* **25**, 1248-1253 (2013).

5 Vargo, K. B., Parthasarathy, R. & Hammer, D. A. Self-assembly of tunable protein suprastructures from recombinant oleosin. *Proceedings of the National Academy of Sciences* **109**, 11657-11662 (2012).

6 Haynes, W. M. *CRC handbook of chemistry and physics*. (CRC press, 2014).

7 Luong, T. K. N. *et al.* Multinuclear diffusion NMR spectroscopy and DFT modeling: A powerful combination for unraveling the mechanism of phosphoester bond hydrolysis catalyzed by metal-substituted polyoxometalates. *Chemistry–A European Journal* **21**, 4428-4439 (2015).

8 Larhed, A. W., Artursson, P., Gråsjö, J. & Björk, E. Diffusion of drugs in native and purified gastrointestinal mucus. *Journal of pharmaceutical sciences* **86**, 660-665 (1997).
9 Sengupta, S. *et al.* Self-powered enzyme micropumps. *Nat Chem* **6**, 415-422 (2014). https://doi.org:10.1038/nchem.1895
10 Kruzel, M. & Morawiecka, B. Acid phosphatase of potato tubers (Solanum tuberosum L). Purification, properties, sugar and amino acid composition. *Acta biochimica Polonica* **29**, 321-330 (1982).
11 Ortiz-Rivera, I., Shum, H., Agrawal, A., Sen, A. & Balazs, A. C. Convective flow reversal in self-powered enzyme micropumps. *Proceedings of the National Academy of Sciences* **113**, 2585-2590 (2016).
12 Maiti, S., Shklyaev, O. E., Balazs, A. C. & Sen, A. Self-Organization of Fluids in a Multienzymatic Pump System. *Langmuir* **35**, 3724-3732 (2019). https://doi.org:10.1021/acs.langmuir.8b03607
